# Bridging the Scientific Knowledge Gap and Reproducibility: A Survey of Provenance, Assertion and Evidence Ontologies

**DOI:** 10.1145/3701716.3715483

**Published:** 2025-05-23

**Authors:** Tek Raj Chhetri, Yaroslav O. Halchenko, Dorota Jarecka, Puja Trivedi, Satrajit S. Ghosh, Patrick Ray, Lydia Ng

**Affiliations:** McGovern Institute for Brain Research, Massachusetts Institute of Technology, Cambridge, MA, USA; Department of Psychological and Brain Sciences, Dartmouth College, Hanover, NH, USA; McGovern Institute for Brain Research, Massachusetts Institute of Technology, Cambridge, MA, USA; McGovern Institute for Brain Research, Massachusetts Institute of Technology, Cambridge, MA, USA; McGovern Institute for Brain Research, Massachusetts Institute of Technology, Cambridge, MA, USA; Allen Institute for Brain Science, Seattle, WA, USA; Allen Institute for Brain Science, Seattle, WA, USA

**Keywords:** Reproducibility, Assertion and Evidence Ontology, Knowledge Graphs, Ontology Survey, Knowledge Discovery and Integration, Provenance

## Abstract

The rapid growth of scientific publications and evolving experimental paradigms create significant challenges in staying up-to-date with current advances. Assertions are often unstructured and have limited provenance, which hinders reproducibility. Ontologies and knowledge graphs (KGs) offer structured solutions by capturing assertions, evidence, and provenance to support reproducibility. This paper reviews 23 ontologies – 13 focused on assertions and evidence and 10 on provenance – providing an overview of the current landscape while highlighting key challenges and opportunities for improvement.

## 1 Introduction

Today, scientific output is expanding at an exponential rate, accelerated by the rapid growth in publications generation. For example, in October 2024 alone, arXiv^1^ saw 24,226^2^ new submissions, while bioRxiv^3^ received 5,222^4^ submissions. Similarly, comparable figures can be observed across platforms, such as medRxiv^5^ and major publishing houses, highlighting the ever-growing volume of scientific outputs across disciplines. Each submission presents assertions, accompanied by supporting evidence, which are essential components for advancing scientific innovation and discovery. However, the surge in research output, coupled with the manner in which findings are reported, presents two major challenges: (i) it is difficult for any individual to keep up with the latest advancements, i.e., what assertions were made and what were the supporting evidence; and (ii) some of the findings might not be reproducible.

At the same time, the scientific community is grappling with a reproducibility crisis, marked by difficulties in consistently reproducing research results. A study by Baker [1] reveals that *70% of researchers have been unable to replicate experiments conducted by other scientists, and more than 50% have faced challenges reproducing their own work*. Similarly, *90% of scientists acknowledge the existence of the reproducibility crisis* [7]. This crisis arises due to: (i) lack of detailed documentation of evidence, such as methodologies or data used [2] and the assertions made; and (ii) inadequate provenance information, including details of intermediate data processing steps, and the processes by which data was collected [6]. Irreproducibility leads to limited return from significant time and financial investments. For example, studies [3] estimate that *irreproducible research in the life sciences alone incurs an annual cost of approximately $28 billion* in the United States. Addressing the reproducibility challenge requires thorough documentation of provenance, evidence, and associated assertions in a manner that aligns with the FAIR (Findable, Accessible, Interoperable, and Reusable) principles. “Thorough” refers to ensuring sufficient details for reliable reproducibility. In addition, recording the assertions, evidence, and provenance helps researchers stay up-to-date with the latest advances amid the exponential growth in publications.

Semantic web technology, particularly ontologies and knowledge graphs (KGs), provides structured and formalized representations of scientific data and findings, and also aligns with the FAIR principles. Ontologies and KGs provide a framework for data representation, sharing, and integration, thereby strengthening each dimension of FAIR and enhancing overall data accessibility and reusability. Most notably, they connect entities through defined relationships, are computable, and supports complex queries, facilitating knowledge discovery. Ontologies and KGs can help connect assertions, evidence, and provenance, and thus provide a comprehensive view to researchers connecting assertions with evidence, and describing the provenance—from the initial step (e.g., data collection) to the final analysis. This structured representation and recording of information can help researchers stay updated on recent developments but also fosters reproducibility. Efforts have therefore focused on constructing ontologies to capture assertions, evidence, and provenance. However, there is no overview of the landscape of such ontologies. This paper aims to address this gap and answer the following research questions (RQs):

RQ1: What existing ontologies support the representation of assertions, evidence, and provenance for scientific experiments (or study)?

RQ2: If such ontologies exist, in which domains are they applied, and what are their characteristics?

## 2 Related Work

Several studies have reviewed provenance. For example, Herschel et al. [5] provide an overview of the provenance research landscape, such as discussing forms and types of provenance for different applications. Similarly, Simmhan et al. [8] provide a survey of various data provenance techniques and propose a taxonomy to classify them. Gierend et al. [4] study provenance tracking in the biomedical domain. However, to the best of our knowledge, no reviews have specifically focused on provenance ontologies. The same is true of ontologies for assertion and evidence. This makes our work the first of its kind to offer a comprehensive overview of ontologies that facilitate capturing provenance in scientific experiments and recording assertions and evidence.

## 3 Methodology

**Figure 1:**
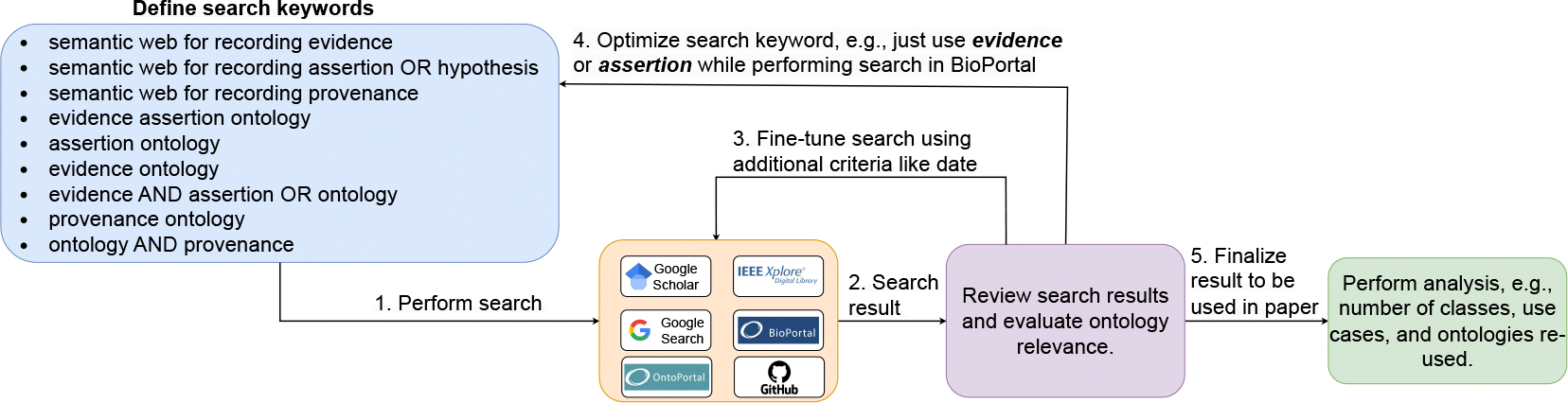
Overview of process for ontology collection and analysis

Figure 1 illustrates the methodology used to collect the relevant ontologies for our study. The process begins with defining the search criteria, which then guide the search process across platforms. Our primary sources include Google Scholar^6^ and IEEE Xplore^7^. Additionally, we used Google and GitHub search, and specialized ontology portals: BioPortal^8^ and OntoPortal^9^. After initial search results, a review was performed to determine the ontological relevance. This involved evaluating whether the ontology captures essential elements such as assertions, evidence, and provenance in scientific publications and experiments. For example, while an Emission Calculations ontology^10^ records the provenance data, it focuses on emissions rather than experimental data and thus does not meet our criteria and is excluded from our study. Similarly, GDPRov^11^ focuses on the provenance of the GDPR, i.e., activities involving the consent and personal data rather than the provenance of scientific experiments, thus it is excluded from our study. Further refinement was performed by adjusting the criteria, such as publication date and keyword variations^12^, to enhance the search results. Finally, analysis, such as analyzing the number of classes and reused ontologies, is performed on the selected ontologies. The selected ontologies were further analyzed to identify reused components by examining the namespaces used in the ontology, reviewing descriptions provided in relevant publications, and looking at the available ontology documentation. It is important to note that ontology search was challenging as some were not easily findable due to hosting heterogeneity. For example, EXPO is on SourceForge^13^, and SWAN, previously on Google Code^14^, was rescued and made available on GitHub. Additionally, some ontologies, like ARGO, were located through their published papers, whereas others, such as AMO, lacked similar references for lookup.

## 4 Ontologies related to assertion and evidence Ontology

Table 1 shows the list of the ontologies that allow recording of the assertions or evidence. It also highlights additional characteristics of these ontologies, including the number of classes, data properties, object properties, domain covered by the ontology, and reused ontologies (or vocabularies). Although the ontologies listed in Table 1 enable the representation of assertions and evidence, not all of them explicitly model the concepts of assertions and evidence. For example, AMO and ARGO ontologies use the *“Argument”* class to capture assertion while the SEPIO and RDO ontologies explicitly model the concept of the *“Assertion”*. SIO ontology employs the concept of *“Argument”* to represent assertion and uses classes^15^ like *“Evidence”* and *“Justification”* to substantiate the assertions. In contrast to AMO and SIO, in RDO, the *“Argument”* class is used to represent a series of assertions. The class *“Claim”* in SWAN ontology can be used to represent an assertion. ECO defined the assertion method, manual and automatic, to record the assertion. The Nanopublication ontology defines the *“AssertionTemplate”* class, which enables the description of assertions within nanopublications. Unlike SE-PIO and ECO, which include an *“Evidence”* (or *“EvidenceItem”* in SEPIO) class for recording evidence to support assertions, nanopublications do not explicitly define such a class. Instead, they rely on provenance information to describe how an assertion was derived, such as linking to publications that contributed to its generation. Similar to SWAN, EVI uses *“Claim”* to represent assertions. However, EVI does not explicitly define an evidence class. Instead, it defines a *“Reference”* class, which can serve as evidence in a manner similar to ECO. The evidence ontology focuses specifically on modeling evidence that supports assertions, categorizing various types of evidence such as experimental findings, curator inferences, and statements from authors in publications. The other ontologies, HOH, OBI, and EXPO, do not explicitly define dedicated classes for assertions and evidence but can still be utilized to record such information. For instance, HOH includes a *“Hypothesis”* class that can represent assertions. OBI features the *“Information Content Entity”* class, also present in ECO, which can be used for capturing evidence. Similarly, EXPO offers classes that support the representation of hypothesis and results, which can be used to model both assertions and evidence effectively.

## 5 Ontologies related to provenance

Table 2 shows the list of ontologies that allows capturing of the provenance of the scientific experiments. These ontologies enable the recording of information such as data sources, experimental procedures, and algorithms utilized in the process. Similar to the ontologies for assertions and evidence, most of these ontologies focus on the life sciences domain. However, a few exceptions, namely RVO, PAV, OPMW and PROV, are domain-agnostic ontologies. RVO defines classes such as *“DataSource”* and *“Dataset”*, along with properties like *“independentVariable”* and *“controlVariable”*, making it applicable to domains beyond the life. Similarly, the PAV ontology defines properties such as *“hasVersion”*, *“hasCurrentVersion”*, and *“providedBy”*, which can be applied across various domains. Likewise, the OPMW ontology introduces classes such as *“Work-flowExecutionProcess”* and *“ParameterVariable”*, which can be used to model scientific workflows across domains. PROV, a W3C recommendation, is a general-purpose provenance ontology applicable across all domains. As shown in Table 2, many ontologies that aim to capture provenance in specific, narrower domains reuse PROV as a foundational model. However, there are exceptions, such as the PROPREO ontology, which does not incorporate PROV. Instead, it focuses on capturing domain-specific provenance using classes like *“HPLC_operating_parameter_collection”*.

## 6 Discussion

The significance of scientific assertions, evidence, and their provenance has been recognized by numerous studies and researchers, leading to the development of various ontologies for formalizing these concepts. This has also led to the different ongoing efforts in both academia and industry, such as Nanopublications^16^, CFDE workbench^17^, Discourse Graphs^18^, and BrainKB^19^, which leverage formalized ontologies to promote FAIR scientific results and ensure robust provenance for reproducibility. Our survey revealed following key issues: (1) *sustainability* – many ontologies were not easily accessible or findable, limiting their reusability; (2) *lack of vocabulary integration* – despite existing standards, they are often not utilized (For instance, while PROPREO models the concept of an agent, it does not leverage the equivalent agent class from the PROV ontology, despite sharing the same interpretation); and 3) *lack of harmonization (or standardization)* – particularly for assertion and evidence ontologies. While provenance ontologies widely reuse the standardized PROV model, assertion and evidence ontologies lack such standardization, as observed in Section 4. We argue for a general, standardized ontology for assertions and evidence, similar to PROV.

## 7 Conclusion & future outlook

In conclusion, our analysis identifies 13 ontologies supporting the representation of assertions and evidence and 10 ontologies focused on provenance in scientific experiments, demonstrating their application across diverse domains, answering RQ1. Sections 4 and 5 summarize their characteristics (RQ2), highlighting classes, properties, reused vocabularies and domain-specific adoption. These findings show the progress and gaps in ontology development for scientific knowledge representation. Future work should focus on addressing the key challenges outlined in Section 6 and also developing essential tools that leverage and reuse existing formalized ontologies. Additionally, future work includes analyzing ontology usage, such as adoption rates, to gain deeper insights and identify trends.

## Supplementary Material

teaser video

## Figures and Tables

**Table 1:** List of ontologies that can be used for (or allows) recording of the assertions or evidence.

Ontology	Class/ Individual	Object/Data Property	Language	Domain	Reused ontologies/vocabularies

ECO	3337/18	47/9	OWL	Biological research	GO, OBI, OIO, CHEBI, DC, SO, NCBITAXON, PR, Dublin Core, IDO, UBERON, IAO, PATO, BFO, RO, COB, UO, CHMO, CL
SIO	1584/0	211/1	OWL	Biomedical	Dublin Core, FOAF, CiTO
RDO	15/0	36/1	OWL	Biomedical	Dublin Core
SEPIO	125/21	210/32	OWL	Generic	BFO, COB, GO, IAO, OBI, RO, Dublin Core, OBI, PATO, STATO, PROV-O, FOAF
AMO	8/0	21/0	OWL	-	CiTO
ARGO	13/8	20/0	OWL	Generic	IAO, BFO, INFO
EXPO	324/0	78/0	OWL	Generic	-
OBI	4924/304	85/5	OWL	Biomedical	CHEBI, GO, DC NCBITAXON, OMRSE, UBERON, VO, IAO, SO, PR, DCTERMS, CLO, OMO, OGMS, HP, IDO, OPL, OIO, OBA, PATO, CL, BFO, RO, COB, ENVO, UO, CHMO, MRO
Nanopublication	21/3	9/6	OWL	Generic	Dublin Core, SKOS, VANN
SWAN	*	*	OWL	Neuromedicine	
HOH	468/18	194/4	OWL	Biology	Dublin Core, CCON, NCI Thesaurus, BFO, ENVO, IAO, RO, Taxslim, EFO
Evidence ontology	40/0	0/4	OWL	Genomics	vCard, Dublin Core, DAML
EVI	30/3	39/21	OWL	Biomedical	PROV-O, Schema.org, VANN, SWRLA, Dublin core, Bioschemas

* represent multiple sub-ontologies and therefore number of classes and properties were not calculated. Details about the reused ontologies can be found at https://doi.org/10.5281/zenodo.14879204. OWL = Web Ontology Language.

**Table 2:** List of provenance ontologies enabling the documentation of task provenance in scientific experiments or studies. Details about the reused ontologies can be found at https://doi.org/10.5281/zenodo.14879204.

Ontology	Class/ Individual	Object/Data Property	Language	Domain	Reused ontologies/vocabularies

RVO	12/5	17/4	OWL	Research data analysis (tools & techniques)	FaBiO, DBPedia, Dublic core, FOAF
PAV	2/9	29/11	OWL	Scientific and digital content	PROV, FOAF, Dublin core
OPMW (or OPMW-PROV)	12/0	10/16	OWL	Scientific workflows	P-Plan, PROV, OPMO, FOAF, Dublin core, OPMV
PROV	51/6	60/9	OWL	Generic	-
P-Plan	10/1	9/0	OWL	Scientific processes	PROV, VANN, Dublin Core
PMLM	19/0	16/4	OWL	Machine learning	PROV
NeuroBridge	667/4	42/6	OWL	Neuroimaging	PROV, PROVCARE, SNOMEDCT
HED	1358/0	14/32	OWL	Neuroimaging and behavioral experiments	Dublin core, FOAF
REPRODUCE-ME	309/249	15/10	OWL	Scientific experiments	PROV, P-Plan
PROPREO	398/0	32/1	OWL	Proteomics	DAML, Dublin core

## CCS Concepts

• **Information systems** → **Ontologies**; **• Computing methodologies** → **Ontology engineering**; *Semantic networks*; • **General and reference** → *Surveys and overviews*; • **Applied computing** → *Computational biology*.

## Footnotes

1
https://arxiv.org

2
https://arxiv.org/stats/monthly_submissions

3
https://www.biorxiv.org

4 The authors manually retrieve the number from bioRxiv.

5
https://www.medrxiv.org

6
https://scholar.google.com

7
https://ieeexplore.ieee.org/Xplore/home.jsp

8
https://bioportal.bioontology.org

9
https://ontoportal.org

10
https://github.com/TEC-Toolkit/PECO

11
https://www.w3.org/community/dpvcg/wiki/GDPRov

12 For example, keyword variations like “prov”, “reproduce”, “evidence ontology github”, “evidence”, and “assertion” were utilized either independently or in combination.

13
https://sourceforge.net/projects/expo

14
https://code.google.com/archive/p/swan-ontology/downloads

15 When referring to classes or properties, we use their labels for readability in case the ontologies employ different encoding systems. For example, in SIO, the class evidence is labeled as SIO_001394, where “*Evidence*” serves as the human-readable label.

16
https://nanopub.net

17
https://info.cfde.cloud/documentation/KGAssertions

18
https://www.discoursegraphs.com

19
https://docs.brainkb.org
